# Adult Day Services as an Essential Service and Support

**DOI:** 10.1093/geroni/igab046.424

**Published:** 2021-12-17

**Authors:** Joseph Gaugler, Katherine Marx, Holly Dabelko-Schoeny, Lauren Parker, Keith Anderson, Elizabeth Albers, Laura Gitlin

**Affiliations:** 1 University of Minnesota, Minneapolis, Minnesota, United States; 2 Johns Hopkins School of Nursing, Baltimore, Maryland, United States; 3 The Ohio State University, The Ohio State University, Ohio, United States; 4 Johns Hopkins Bloomberg School of Public Health, Baltimore, Maryland, United States; 5 University of Texas at Arlington, Arlington, Texas, United States; 6 Drexel University, College of Nursing and Health Professions, Drexel University, Pennsylvania, United States

## Abstract

Throughout the COVID-19 pandemic, the significant challenges and gaps related to the care of older people in the U.S. were made distressingly apparent. This summary presentation will consider the effects of COVID-19 and associated shutdowns on older persons who use ADS programs, their family caregivers, and programs/staff themselves. Among recommendations to consider are the classification of adult day services and similar community-based long-term care providers as essential (and clarifying their difference from senior centers). In addition, considering new financing approaches and utilizing ADS or similar community-based programs as incubators of evidence-based innovation are options to consider to better align ADS with optimal dementia care.

